# Do People Taking Flu Vaccines Need Them the Most?

**DOI:** 10.1371/journal.pone.0026347

**Published:** 2011-12-02

**Authors:** Qian Gu, Neeraj Sood

**Affiliations:** 1 KNG Health Consulting. Rockville, Maryland, United States of America; 2 Schaeffer Center for Health Policy and Economics, University of Southern California, Los Angeles, California, United States of America; Statens Serum Institute, Denmark

## Abstract

**Background:**

A well targeted flu vaccine strategy can ensure that vaccines go to those who are at the highest risk of getting infected if unvaccinated. However, prior research has not explicitly examined the association between the risk of flu infection and vaccination rates.

**Purpose:**

This study examines the relationship between the risk of flu infection and the probability of getting vaccinated.

**Methods:**

Nationally representative data from the US and multivariate regression models were used to estimate what individual characteristics are associated with (1) the risk of flu infection when unvaccinated and (2) flu vaccination rates. These results were used to estimate the correlation between the probability of infection and the probability of getting vaccinated. Separate analyses were performed for the general population and the high priority population that is at increased risk of flu related complications.

**Results:**

We find that the high priority population was more likely to get vaccinated compared to the general population. However, within both the high priority and general populations the risk of flu infection when unvaccinated was negatively correlated with vaccination rates (r = −0.067, p<0.01). This negative association between the risk of infection when unvaccinated and the probability of vaccination was stronger for the high priority population (r = −0.361, p<0.01).

**Conclusions:**

There is a poor match between those who get flu vaccines and those who have a high risk of flu infection within both the high priority and general populations. Targeting vaccination to people with low socioeconomic status, people who are engaged in unhealthy behaviors, working people, and families with kids will likely improve effectiveness of flu vaccine policy.

## Introduction

Seasonal influenza is associated with a large number of hospitalizations and excess deaths in the United States [1]–[3]. Annual vaccination is the most effective strategy for preventing influenza infection. The Advisory Committee on Immunization Practices (ACIP) recommends influenza vaccination for all people age 6 months and older, with a focus on priority populations with a high risk of complications (e.g. older adults, people with certain medical conditions, and pregnant women) and those that come in frequent contact with these populations (e.g. healthcare professionals) [4]. Despite these recommendations, influenza vaccination coverage is still suboptimal. In the 2009–2010 flu season, the national seasonal influenza vaccination coverage among the adult population was 36%, ranging from 28% for adults 18–49 years old without high risk of complications to 68% for adults aged 65 and older [5].

The emergence of new influenza subtypes and antigenic evolution of influenza requires annual updates of the influenza vaccine, which sets a tight schedule for manufacturers to produce sufficient doses. A delay in the schedule can cause shortage of influenza vaccines available for distribution [6]. Contaminated flu vaccines caused a vaccine shortage in the U.S. in the 2004–2005 flu season [7]. More recently, the U.S. faced a shortage of seasonal flu vaccine as well as swine flu vaccine in the 2009–2010 flu season as the H1N1 pandemic raised the demand of flu vaccines well beyond the manufacturers' production capacity [8]. In a flu vaccine shortage, people who need a vaccine may not be able to obtain one. An optimal flu vaccine distribution strategy is imperative to make sure the limited vaccines go to those who need them most.

Several prior studies have examined the correlates of flu vaccination. The vast majority of this literature has focused on priority populations such as the elderly and healthcare workers. Significant predictors of flu vaccination among priority populations include perceived effectiveness and safety of vaccines, as well as beliefs about own health status and risk of infection [9]–[18]. There is concern that some of these beliefs might be mistaken. For example, some consumers have the mistaken assumption that healthy people do not need immunization, while others believe that vaccination can cause serious side effects [10]–[11], [15], [17], [19]–[23]. Important barriers to vaccination include lack of insurance and lack of physician recommendation for vaccinations [9], [11]–[15], [21]–[25]. Several studies also document racial disparities in vaccination coverage with African Americans and Hispanics having lower vaccination rates compared to Whites [15], [21], [24], [26]–[38]. Some studies have also shown that those with unhealthy lifestyles are less likely to be vaccinated [39]–[41].

There are only a few studies that examine the correlates of flu vaccination in the general population. One study using a national sample of adults from the U.S. aged 50–64 years found that previous doctor visits, education and perceived vaccine effectiveness and safety were important predictors of flu coverage [42]. Two studies using a national sample of adults from Canada and Korea found that physician or nurse recommendation was the major predictor of vaccination [43]–[44]. Other studies on vaccination coverage among the general population have relied on much smaller samples from a few employers or communities [45]–[47].

While the prior literature documents some important predictors of flu vaccination, it does not explicitly examine whether those receiving the flu vaccine have a higher risk of infection were they not vaccinated. A positive association between risk of infection and vaccination would suggest that the current vaccination program is well targeted. Conversely, a negative association would suggest poor targeting. Thus, examining the association between risk of infection and vaccination is an important metric for evaluating the effectiveness of the current flu vaccination program. This information is also important for developing an optimal vaccination strategy, especially given the vaccine shortages experienced in recent flu seasons and the suboptimal vaccination rate among both the general and high priority populations. In this paper, we addresses this gap in the literature by using nationally representative data from the U.S. to examine the association between risk of flu infection when unvaccinated and vaccination rates among both the general and high priority populations.

## Methods

### Data

The study used data from the 2007 National Health Interview Survey (NHIS) to examine the correlates of flu vaccination and flu infection. NHIS is a cross-sectional household interview survey that uses a national representative sample of the civilian noninstitutionalized population of the United States. The sample for analysis was restricted to sample adults aged 18 years and older. The analysis used the 2007 wave of NHIS for analysis because it is the only wave that asked about both flu vaccination and infection (other waves have asked about flu vaccination only).

### Outcome Measures

Respondents in the 2007 NHIS were asked “Have you ever been told by a doctor or other health professional that you had influenza or pneumonia?” If the answer is yes, the respondents were asked “During the past 12 months, have you had influenza or pneumonia?” Respondents were coded as having had the flu in the past 12 months if they answered yes to both of the above two questions. Flu vaccination status was determined by the two following questions: “During the past 12 months, have you had a flu shot? A flu shot is usually given in the fall and protects against influenza for the flu season.” and “During the past 12 months, have you had a flu vaccine sprayed in your nose by a doctor or other health professional? A health professional may have let you spray it. This vaccine is usually given in the fall and protects against influenza for the flu season. This influenza vaccine is called FluMist.” Respondents were coded as having had a flu vaccine in the past 12 months if they answered yes to either of the two questions.

### Explanatory Variables

Based on prior research several groups of explanatory variables were included in the statistical analysis, including demographics, family composition, health status, health insurance and health behaviors. Demographic variables included gender, age, race and ethnicity, marital status, education and working status, number of adults and number of kids in the family. Health insurance was measured by whether respondents were covered by any health insurance. Health behaviors and measures included self reported health status(poor to excellent), obesity status (underweight, normal weight, overweight, obese), smoking and drinking status, physical exercise, regular strength training, and whether the respondent reported having a regular place to go for preventive medical care. Heavy drinking was defined as over 14/7 (male/female) drinks per week in the past year. Moderate drinking was defined as 1–14/1–7 (male/female) drinks per week in the past year. Respondents were asked about their time spent on rigorous and moderate physical exercise per week. Respondents were coded as physically active (dummy variable set to 1) if they had at least 150 minutes of physical exercise per week (assuming 1 minute of rigorous exercise is equivalent to 2 minutes of moderate exercise based on CDC guidelines [48] ). Respondents were considered to do regular strength training if they had at least one strength training session per week.

### Statistical Analysis

The pattern of flu vaccination and infection could be quite different across low and high priority groups for flu immunization as recommendations from ACIP for high priority groups can potentially influence provider and consumer behavior with regard to flu vaccination. Therefore, a separate statistical analysis was conducted for each of the two groups. Consistent with the CDC definition, people in the high priority group included people aged 65 and older, pregnant women, healthcare workers and anyone with at least one of the following medical conditions: asthma, neurological and neurodevelopmental conditions, COPD/emphysema, diabetes, coronary artery disease, HIV/AIDS, cancer and kidney and liver disorders [49].

For each group the association between risk of flu infection and the probability of receiving flu vaccination was examined. The analysis sample for examining the risk of flu infection was restricted to respondents who did not receive a flu vaccine in the past 12 months. The analysis started by comparing the average rate of infection and vaccination by key explanatory variables and used one-way Analysis of Variance (ANOVA) to test the equality of infection and vaccination rate across different groups. Next, multivariate regression analysis was used to test if the associations documented in the initial comparison of means were robust to inclusion of multiple covariates. Ideally, one wants to observe flu infection status for everyone in the sample conditional on not taking the flu vaccine. In reality, however, one cannot observe this counterfactual infection status for people who actually did take flu vaccine. Therefore, studying the risk of infection using infection information from the subsample of respondents who chose not to get vaccinated might produce biased estimates. The reason is that the decision to get vaccinated might be correlated with unobservable factors that influence the risk of infection. To address this selection bias, the analysis used a Heckman Probit model with selection, which jointly estimates the probability of not being vaccinated (the selection equation) and the probability of flu infection (the outcome equation) and allows for both decisions to be correlated based on observable as well as unobservable factors [50]–[51]. The dependent variables in the analysis are indicator variables for whether the respondent was vaccinated in the past 12 months and whether the respondent got flu infection in past 12 months. Separate regressions were estimated for each priority group. The results from these regression models were used to predict the probability of flu infection if unvaccinated and the probability of flu vaccination for each respondent. Finally, the correlation between these probabilities was estimated. The statistical analysis was conducted using Stata SE 11.0 (StataCorp, College Station, TX).

## Results

### Summary Statistics

The final analytic sample had 23,393 adults aged 18 and older. The high priority group accounted for 43 percent of the entire sample. The rest of them were designated as “low priority group”. Table 1 lists the weighted summary statistics of the sample. Compared with the low priority group, people in the high priority group had a substantially higher rate of flu infection (7% vs. 4%). Readers are reminded that the flu infection rate was calculated for people who did not take flu vaccine in the past 12 months. People in the high priority group were more likely to be vaccinated (48% vs. 18%), which is consistent with the ACIP recommendations.

**Table 1:** Summary Statistics of the Analytical Sample.

Variables	Low Priority Group		High Priority Group		Combined	
	Mean	S.D.	Mean	S.D.	Mean	S.D.
Had Flu in Past 12 Months	0.04	0.20	0.07	0.25	0.05	0.22
Had Flu Vaccine in Past 12 Months	0.18	0.38	0.48	0.50	0.30	0.46
Age	38.80	12.74	56.39	18.50	45.79	17.55
Male	0.53	0.50	0.41	0.49	0.48	0.50
Race & Ethnicity: Non-Hispanic White	0.66	0.47	0.75	0.43	0.69	0.46
Race & Ethnicity: Black	0.12	0.32	0.11	0.32	0.12	0.32
Race & Ethnicity: Hispanics	0.16	0.37	0.10	0.29	0.13	0.34
Race & Ethnicity: Non-Hispanic Others	0.06	0.24	0.04	0.21	0.06	0.23
Married	0.63	0.48	0.62	0.49	0.62	0.48
Education: Less than High School	0.14	0.35	0.18	0.39	0.16	0.36
Education: High School Graduate	0.28	0.45	0.30	0.46	0.29	0.45
Education: Some College	0.29	0.45	0.28	0.45	0.28	0.45
Education: College Graduate and Above	0.29	0.45	0.24	0.43	0.27	0.44
Number of Adults in Family	2.14	0.94	2.00	0.84	2.08	0.90
Number of Kids in Family	0.86	1.18	0.46	0.97	0.70	1.12
Worked in the Past 12 Months	0.84	0.36	0.51	0.50	0.71	0.45
Covered by Any Health Insurance	0.79	0.41	0.90	0.30	0.83	0.37
Health: Excellent	0.36	0.48	0.18	0.38	0.29	0.45
Health: Very Good	0.35	0.48	0.27	0.44	0.32	0.47
Health: Good	0.23	0.42	0.31	0.46	0.26	0.44
Health: Fair or Poor	0.06	0.23	0.25	0.43	0.13	0.34
BMI: Normal Weight	0.39	0.49	0.34	0.47	0.37	0.48
BMI: Underweight	0.02	0.13	0.02	0.14	0.02	0.13
BMI: Overweight	0.36	0.48	0.35	0.48	0.35	0.48
BMI: Obese	0.24	0.43	0.30	0.46	0.26	0.44
Use Preventive Medical Care	0.85	0.36	0.94	0.23	0.89	0.32
Smoking: Non-Smoker	0.79	0.41	0.83	0.38	0.80	0.40
Smoking: Light Smoker (Someday Only)	0.05	0.22	0.03	0.18	0.04	0.20
Smoking: Heavy Smoker (Everyday)	0.16	0.37	0.14	0.35	0.15	0.36
Drinking: Non-Drinker	0.33	0.47	0.48	0.50	0.39	0.49
Drinking: Light/Moderate Drinker	0.61	0.49	0.48	0.50	0.56	0.50
Drinking: Heavy Drinker	0.06	0.24	0.04	0.19	0.05	0.22
Physical Exercise: > = 150 Min/Wk	0.44	0.50	0.34	0.47	0.40	0.49
Regular Strength Training	0.26	0.44	0.18	0.39	0.23	0.42
Observations	13,406	9,987	23,393			

Note: Summary statistics were weighted using survey weights. Missing values were excluded from calculations.

### ANOVA Model

Table 2 presents the rate of flu infection and vaccination by select explanatory variables (see table S1 for all explanatory variables). An important observation from table 2 is that many of these variables have significant but divergent effects on infection and vaccination rates. In both priority groups, people with a college degree had a higher rate of vaccination than those less well educated, although they were no more likely to contract flu if unvaccinated. In fact, the college educated had a lower infection rate in the high priority group. People with kids in their family had a higher infection rate but they had a significantly lower vaccination rate. Working people in the high priority group were much less likely to take flu vaccine even though they had a greater risk of infection without a vaccine. In both the high and low priority groups, people with health insurance had a substantially higher vaccination rate (21% vs. 8% in low priority group and 52% vs. 18% in high priority group) but the insured enjoyed a much lower rate of flu infection if unvaccinated. Similarly, people who had a regular place to go for preventive medical care were almost three times more likely to immunize but were less likely to contract flu. Health behaviors were also associated with divergent impacts on flu immunization and infection rates. In both priority groups, heavy smokers were far less likely to vaccinate and more likely to contract flu than non-smokers or light smokers. This trend was especially strong in the high priority group. Heavy drinkers were less likely to get vaccinated in both groups. Heavy drinkers in the high priority group also had a higher flu infection rate. In the low priority group, physically active people (time for physical activities > = 150 min/wk) and people who did regular strength training had a higher rate of vaccination but the same rate of infection, compared with people with a lower level of physical activity. For the high priority group, the difference in infection and vaccination by physical activity level was barely noticeable.

**Table 2:** Percentage of Flu Infection and Vaccination by Select Individual Characteristics.

Variables	Low Priority Group				High Priority Group			
	Flu Infection		Flu Vaccination		Flu Infection		Flu Vaccination	
	%	P	%	P	%	P	%	P
Education: Less Than High School	4.41		13.15		6.92		46.47	
Education: High School Graduate	3.73	0.436	15.44	0.000	6.47	0.170	48.69	0.001
Education: Some College	4.31		17.31		7.73		46.79	
Education: College Graduate and Above	4.53		23.77		5.53		51.80	
2 or Less Adults in Family	4.21	0.818	18.62	0.001	7.10	0.029	50.11	0.000
More Than 2 Adults in Family	4.10		15.99		5.20		40.77	
No Kid in Family	4.02	0.364	20.05	0.000	5.87	0.001	53.33	0.000
At Least One Kid in Family	4.37		15.49		8.51		33.16	
Not Working in the Past 12 Months	4.39	0.591	18.44	0.612	5.67	0.017	57.87	0.000
Working in the Past 12 Months	4.11		17.97		7.41		39.38	
No Health Insurance Coverage	4.93	0.033	7.58	0.000	8.98	0.005	17.61	0.000
Has Health Insurance Coverage	3.96		20.89		6.23		51.73	
Health: Excellent	3.25		18.00		4.06		40.60	
Health: Very Good	4.21	0.000	18.49	0.464	5.20	0.000	49.02	0.000
Health: Good	4.58		17.69		6.80		49.98	
Health: Fair or Poor	8.26		16.21		10.57		51.47	
BMI: Normal Weight	3.50		17.21		5.24		48.09	
BMI: Underweight	6.51	0.018	19.13	0.160	4.80	0.024	44.33	0.022
BMI: Overweight	4.44		18.98		7.39		50.58	
BMI: Obese	4.81		17.89		7.66		46.98	
No Regular Place for Preventive Medical Care	5.22	0.016	7.27	0.000	7.55	0.450	18.43	0.000
Regular Place for Preventive Medical Care	3.98		19.90		6.63		50.33	
Smoking: Non-Smoker	3.70		19.83		5.44		51.84	
Smoking: Light Smoker (Someday Only)	5.16	0.000	13.52	0.000	8.31	0.000	36.54	0.000
Smoking: Heavy Smoker (Everyday)	6.11		10.95		11.48		31.17	
Drinking: Non-Drinker	4.45		18.18		6.36		50.40	
Drinking: Light/Moderate Drinker	4.26	0.399	18.63	0.015	6.72	0.023	47.77	0.000
Drinking: Heavy Drinker	3.28		14.40		11.03		33.75	
Physical Exercise: <150 Min/Wk	4.12	0.741	16.60	0.000	6.75	0.869	48.86	0.223
Physical Exercise: > = 150 Min/Wk	4.25		19.74		6.63		47.57	
No Regular Strength Training	4.17	0.857	17.10	0.000	6.87	0.308	48.07	0.127
Regular Strength Training	4.25		20.64		5.92		50.07	

Note: P indicates P-value associated with one-way ANOVA. Percentage calculations were weighted using survey weights. Only select individual characteristics were reported. See table S1 in supplemental information for a full table.

### Heckman Probit Regression Analysis

Table 3 reports the marginal effects of select explanatory variables on the risk of flu infection when unvaccinated and the probability of flu vaccination from the Heckman probit regression analysis (see table S2 for all explanatory variables in the regression model). The results from the multivariate regression analysis were largely consistent with the results from the univariate analysis reported earlier. In both priority groups, college graduates were significantly more likely to vaccinate compared to those with no high school diploma. However, the risk of infection did not vary significantly by education. People with more kids in their family were significantly more likely to contract the flu but were less likely to get vaccinated. Similarly, working people had a significantly higher infection rate but they had a lower vaccination rate in the high priority group. People covered by health insurance were significantly more likely to vaccinate but did not have a higher risk of flu infection. Those with a regular place to go for preventive medical care were significantly more likely to vaccinate but had a lower risk of infection in the low priority group. All health behavior factors were significant predictors of flu vaccination. Non-smokers were substantially more likely to get flu vaccine and less likely to get flu, compared with the heavy smokers. Similarly, non-drinkers and moderate drinkers were significantly more likely to get vaccinated and they enjoyed a significantly lower risk of infection in the high priority group. Physically active people and people with regular strength training were also significantly more likely to vaccinate, although the effect of physical activities was not significant in the high priority group. There was no significant difference in risk of infection by either physical activity level or strength training level.

**Table 3:** Marginal Effect of Select Factors Associated with Flu Vaccination and Infection.

Variables	Low Priority Group		High Priority Group	
	Infection	Vaccination	Infection	Vaccination
Education [Less Than High School]				
High School Graduate	−0.008	−0.005	−0.005	0.018
	(0.006)	(0.012)	(0.010)	(0.014)
Some College	0.007	0.005	0.004	0.057***
	(0.006)	(0.012)	(0.010)	(0.015)
College Graduate and Above	0.007	0.027**	0.003	0.064***
	(0.007)	(0.012)	(0.011)	(0.016)
Worked in the Past 12 Months	0.009*	0.004	0.014*	−0.025**
	(0.006)	(0.009)	(0.008)	(0.012)
Number of Kids in Family	0.003*	−0.009***	0.007**	−0.012*
	(0.002)	(0.003)	(0.003)	(0.006)
Covered by Any Health Insurance	−0.008	0.091***	−0.015	0.159***
	(0.005)	(0.010)	(0.010)	(0.020)
Regular Place for Preventive Medical Care	−0.012**	0.072***	−0.018	0.158***
	(0.006)	(0.012)	(0.012)	(0.024)
Smoking Status [Current Smoker, Everyday]				
Non-Smoker	−0.016***	0.047***	−0.032***	0.069***
	(0.005)	(0.010)	(0.008)	(0.015)
Current Smoker, Sometimes	−0.009	0.032*	−0.022	0.065**
	(0.009)	(0.018)	(0.017)	(0.029)
Drinking Status [Current Heavy Drinker]				
Current Non-Drinker	0.015	0.032**	−0.025*	0.063**
	(0.009)	(0.016)	(0.015)	(0.026)
Current Moderate Drinker	0.011	0.019	−0.027*	0.088***
	(0.009)	(0.015)	(0.014)	(0.026)
Physical Exercise: > = 150 Min/Week	0.003	0.017**	0.010	0.008
	(0.004)	(0.007)	(0.008)	(0.011)
Strength Training: > = Once/Week	0.004	0.020**	−0.003	0.064***
	(0.005)	(0.008)	(0.010)	(0.014)
Correlation: Pr(Infection) & Pr(Vaccination)	−0.067***		−0.361***	
Observations	10,794	13,078	4,952	9,735

Note: Average marginal effects are reported from Heckman probit selection model. The reported correlation is between predicted probability of flu infection conditional on being not vaccinated and predicted probability of flu vaccination. Group in the brackets is the reference group. Only select variables in the regression model were reported. See table S2 in supplemental information for a full table. Significance:

*p<0.10,

**p<0.05,

***p<0.01.

Other demographics were also significant predictors of flu infection and vaccination. Men had lower odds for both flu infection and vaccination than women. Compared to non-Hispanic Whites, Blacks were significantly less likely to get flu vaccine and to get the flu when unvaccinated. Hispanics were less likely to get vaccinated, however their risk of infection risk was not significantly different from non-Hispanic Whites. Self-reported health status was a significant predictor of flu infection. In contrast with other factors, poor health was associated with both higher risk of flu infection and higher rate of vaccination. Similarly, obese people had a higher probability of both flu infection and vaccination in the high priority group.

### Correlation between Probabilities of Infection and Vaccination

The results from these regression models were used to predict the probability of flu infection when unvaccinated, and the probability of flu vaccination for each respondent. In both priority groups, the risk of flu infection and the probability of vaccination were negatively correlated; and the correlations were significant at 0.01 level. A much stronger negative correlation was observed in the high priority group than the low priority group (−0.36 vs. −0.07). In other words, within each priority group, people who had a higher predicted risk of infection also had a lower predicted probability of vaccination and this trend was much stronger in the high priority group. For example, in the low priority group, the average probability of flu infection was 0.044 for the lowest quartile of the probability of vaccination vs. 0.039 for the highest quartile. In the high priority group, the average probability of flu infection was 0.079 for the lowest quartile of the probability of vaccination vs. 0.040 for the highest quartile.

## Discussion

This study examined the correlates of flu infection and vaccination simultaneously using the same set of explanatory variables. The analysis suggests that, within each priority group, many people with a higher propensity to vaccinate actually had a lower probability of contracting the flu if unvaccinated. Specifically, people with more social resources (higher education, health insurance coverage) and people who took good care of their health (physically active people, non-smokers, non-drinkers and those who use preventive care) were more motivated to protect themselves from flu through vaccination, even though they had a lower risk of infection. In contrast, people with more kids in their family and people who work for pay were at greater risk of infection but were less likely to vaccinate. In general, for each priority group, we need additional programs that target specific population groups that have a higher risk of infection and a lower propensity to vaccinate. Under an optimal flu vaccination policy, one would expect a strong positive correlation between the risk of infection and probability of vaccination, that is, people at a higher risk of infection should have a higher vaccination rate. However, the results from this study suggest that the opposite is true within each priority group: people who had a higher propensity to vaccinate were the same people who were at lower risk of infection. And this mismatch between risk of infection and vaccination rates was much worse in the high priority group, who are more vulnerable to flu-related complications. The only silver lining in the results was that the high priority group had higher vaccination rates compared to the low priority group.

A major strategy to improve flu vaccine allocation reported by previous studies is to vaccinate potential “super-spreaders” like older children and younger adults [52]–[60]. Our analysis concurs with this strategy in that adults with kids living in the family are significantly more likely to get flu infection but less likely to vaccinate. However, our analysis suggests that having kids in the family is just one of the many factors that have divergent effects on flu vaccination and flu infection. While vaccinating older children and younger adults can potentially reduce the source of infection, many people at higher risk of flu infection are still not vaccinated.

Overall the empirical evidence presented in this paper suggests several other ways to address and improve the effectiveness of current flu vaccination programs. First, we should make efforts to improve the vaccination rate among people with low socioeconomic status and few social resources such as minorities, people without insurance coverage and the less educated. Second, we should have an outreach program to target people who are engaged in unhealthy behaviors such as heavy smokers, heavy drinkers, and people with sedentary lifestyles. Third, we should encourage everyone in families with kids to vaccinate. Finally, we should improve flu vaccination at the workplace. However, targeting people who need flu vaccination the most may be a difficult endeavor. Alternatively, a universal flu vaccination program that offers free flu vaccines to all individuals can improve vaccination rate, as demonstrated by evidence from the universal flu immunization program introduced in Ontario, Canada in 2000 [61]. However, a universal flu immunization program may produce disparate impacts on different population groups. The program may effectively boost the flu vaccine uptake in people who are unvaccinated for cost reasons, such as low-income or uninsured population. But the effectiveness of such a program among people with unhealthy lifestyles is questionable. Unhealthy lifestyles often reveal people's attitude toward risk-taking and preference for investments in health, which are unlikely to be responsive to a lower cost of flu vaccine. For this group, a targeted vaccine program that includes both subsidies and educational and behavioral interventions may be necessary. Comparing the effectiveness of each of these and other strategies for targeting flu vaccines is an important topic for future research. Additionally, future research can build upon this analysis and explore the impact of other factors on both flu infection and vaccination, such as adoption of other preventive behaviors during the flu season (e.g. washing hands more frequently, avoiding contact with people with flu-like symptoms and avoiding public transportation or other crowded places), preferences or attitudes towards risks, influence of peers, and cultural attitudes towards modern medicine and disease processes.

The findings of this study should be viewed in light of its limitations. Although the study uses nationally representative data, the data have some shortcomings. First, flu infections were self-reported. The study was not able to verify and measure the level of misreporting as the official annual flu season summary prepared by CDC only reports the weekly percentage of patient visits to physicians for influenza-likely illness [62]. Second, the survey was conducted continuously during the year 2007, which means the time period of “the past 12 months” varies depending on the time of the survey. Third, the flu infection data is available only in the 2007 NHIS survey. Ideally, one would like to study flu vaccination and infection in other years, especially those years with a flu pandemic and a flu vaccine shortage. Lastly, as with most population-based surveys, we can only use NHIS to track “influenza like illness”. There are two concerns with using this measure to estimate the correlates of flu infection if unvaccinated. First, in principle influenza like illness can be caused by several viruses including the influenza virus. Second, influenza like illness does not capture infection risk for individuals who only suffer from asymptomatic flu infection. However, we believe that these are not significant concerns for several reasons. Prior research shows that diagnosis based on influenza like symptoms is highly correlated with laboratory-confirmed influenza infection [63]. Prior research also shows that as much as two thirds of all flu infections are symptomatic and suggests that the risk of symptomatic flu infection is highly correlated with the overall risk of flu infection [64]. In addition, we are not aware of any prior research that highlights any socioeconomic or demographic factors that predict the risk of symptomatic flu infection but not asymptomatic infection. Finally, there is little research that establishes the infectivity of individuals with asymptomatic infection. However, evidence from studies among individuals with symptomatic infection show that the infectivity of individuals as measured by viral shedding is highly positively correlated with the severity of symptoms [65]–[66].

## Supporting Information

Table S1 Percentage of Flu Infection and Vaccination by Individual Characteristics.
(DOC)

Table S2 Marginal Effect of Factors Associated with Flu Vaccination and Infection.
(DOC)
