# Supplementary material for: Do People Taking Flu Vaccines Need Them the Most?
Source: PLoS One. 2011 Dec 2;6(12):e26347. doi: 10.1371/journal.pone.0026347 (PMC3229476; doi:10.1371/journal.pone.0026347)
Supplement: Table S1 — Percentage of Flu Infection and Vaccination by Individual Characteristics. (DOC) [file pone.0026347.s001.doc]

| **Table S1 : Percentage of Flu Infection and Vaccination by Individual Characteristics** | | | | | | | | |
| --- | --- | --- | --- | --- | --- | --- | --- | --- |
|  |  |  |  |  |  |  |  |  |
| Variables | Low Priority Group | | | | High Priority Group | | | |
|  | Flu Infection | | Flu Vaccination | | Flu Infection | | Flu Vaccination | |
|  | % | P | % | P | % | P | % | P |
| Male | 3.95 | 0.201 | 16.61 | 0.000 | 6.18 | 0.217 | 47.86 | 0.369 |
| Female | 4.45 |  | 19.58 |  | 7.07 |  | 48.79 |  |
| Race and Ethnicity: Non-Hispanic White | 4.53 |  | 20.05 |  | 7.36 |  | 51.55 |  |
| Race and Ethnicity: Non-Hispanic Black | 1.85 | 0.000 | 13.02 | 0.000 | 3.48 | 0.002 | 37.64 | 0.000 |
| Race and Ethnicity: Hispanics | 4.93 |  | 12.32 |  | 7.21 |  | 36.45 |  |
| Race and Ethnicity: Non-Hispanic Others | 3.26 |  | 20.32 |  | 4.89 |  | 48.69 |  |
| Married | 4.47 | 0.069 | 20.07 | 0.000 | 6.74 | 0.919 | 48.88 | 0.267 |
| Not Married | 3.75 |  | 14.52 |  | 6.66 |  | 47.73 |  |
| Education: Less Than High School | 4.41 |  | 13.15 |  | 6.92 |  | 46.47 |  |
| Education: High School Graduate | 3.73 | 0.436 | 15.44 | 0.000 | 6.47 | 0.170 | 48.69 | 0.001 |
| Education: Some College | 4.31 |  | 17.31 |  | 7.73 |  | 46.79 |  |
| Education: College Graduate and Above | 4.53 |  | 23.77 |  | 5.53 |  | 51.80 |  |
| 2 or Less Adults in Family | 4.21 | 0.818 | 18.62 | 0.001 | 7.10 | 0.029 | 50.11 | 0.000 |
| More Than 2 Adults in Family | 4.10 |  | 15.99 |  | 5.20 |  | 40.77 |  |
| No Kid in Family | 4.02 | 0.364 | 20.05 | 0.000 | 5.87 | 0.001 | 53.33 | 0.000 |
| At Least One Kid in Family | 4.37 |  | 15.49 |  | 8.51 |  | 33.16 |  |
| Not Working in the Past 12 Months | 4.39 | 0.591 | 18.44 | 0.612 | 5.67 | 0.017 | 57.87 | 0.000 |
| Working in the Past 12 Months | 4.11 |  | 17.97 |  | 7.41 |  | 39.38 |  |
| No Health Insurance Coverage | 4.93 | 0.033 | 7.58 | 0.000 | 8.98 | 0.005 | 17.61 | 0.000 |
| Has Health Insurance Coverage | 3.96 |  | 20.89 |  | 6.23 |  | 51.73 |  |
| Health: Excellent | 3.25 |  | 18.00 |  | 4.06 |  | 40.60 |  |
| Health: Very Good | 4.21 | 0.000 | 18.49 | 0.464 | 5.20 | 0.000 | 49.02 | 0.000 |
| Health: Good | 4.58 |  | 17.69 |  | 6.80 |  | 49.98 |  |
| Health: Fair or Poor | 8.26 |  | 16.21 |  | 10.57 |  | 51.47 |  |
| BMI: Normal Weight | 3.50 |  | 17.21 |  | 5.24 |  | 48.09 |  |
| BMI: Underweight | 6.51 | 0.018 | 19.13 | 0.160 | 4.80 | 0.024 | 44.33 | 0.022 |
| BMI: Overweight | 4.44 |  | 18.98 |  | 7.39 |  | 50.58 |  |
| BMI: Obese | 4.81 |  | 17.89 |  | 7.66 |  | 46.98 |  |
| No Regular Place for Preventive Medical Care | 5.22 | 0.016 | 7.27 | 0.000 | 7.55 | 0.450 | 18.43 | 0.000 |
| Regular Place for Preventive Medical Care | 3.98 |  | 19.90 |  | 6.63 |  | 50.33 |  |
|  |  |  |  |  |  |  |  |  |
| **Table S1 : Percentage of Flu Infection and Vaccination by Individual Characteristics (Cont'd)** | | | | | | | | |
|  |  |  |  |  |  |  |  |  |
| Variables | Low Priority Group | | | | High Priority Group | | | |
|  | Flu Infection | | Flu Vaccination | | Flu Infection | | Flu Vaccination | |
|  | % | P | % | P | % | P | % | P |
| Smoking: Non-Smoker | 3.70 |  | 19.83 |  | 5.44 |  | 51.84 |  |
| Smoking: Light Smoker (Someday Only) | 5.16 | 0.000 | 13.52 | 0.000 | 8.31 | 0.000 | 36.54 | 0.000 |
| Smoking: Heavy Smoker (Everyday) | 6.11 |  | 10.95 |  | 11.48 |  | 31.17 |  |
| Drinking: Non-Drinker | 4.45 |  | 18.18 |  | 6.36 |  | 50.40 |  |
| Drinking: Light/Moderate Drinker | 4.26 | 0.399 | 18.63 | 0.015 | 6.72 | 0.023 | 47.77 | 0.000 |
| Drinking: Heavy Drinker | 3.28 |  | 14.40 |  | 11.03 |  | 33.75 |  |
| Physical Exercise: < 150 Min/Wk | 4.12 | 0.741 | 16.60 | 0.000 | 6.75 | 0.869 | 48.86 | 0.223 |
| Physical Exercise: >= 150 Min/Wk | 4.25 |  | 19.74 |  | 6.63 |  | 47.57 |  |
| No Regular Strength Training | 4.17 | 0.857 | 17.10 | 0.000 | 6.87 | 0.308 | 48.07 | 0.127 |
| Regular Strength Training | 4.25 |  | 20.64 |  | 5.92 |  | 50.07 |  |
| Note: P indicates P-value associated with one-way ANOVA. Percentage calculations were weighted using survey weights. | | | | | | | | |
|
