# Supplementary material for: Do People Taking Flu Vaccines Need Them the Most?
Source: PLoS One. 2011 Dec 2;6(12):e26347. doi: 10.1371/journal.pone.0026347 (PMC3229476; doi:10.1371/journal.pone.0026347)
Supplement: Table S2 — Marginal Effect of Factors Associated with Flu Vaccination and Infection. (DOC) [file pone.0026347.s002.doc]

| **Table S2: Marginal Effect of Factors Associated with Flu Vaccination and Infection** | | | | |
| --- | --- | --- | --- | --- |
|  |  |  |  |  |
| Variables | Low Priority Group | | High Priority Group | |
|  | Infection | Vaccination | Infection | Vaccination |
| Male | -0.007* | -0.021*** | -0.019*** | -0.039*** |
|  | (0.004) | (0.007) | (0.007) | (0.010) |
| Age | 9.46E-5 | 0.004*** | -4.73E-4** | 0.007*** |
|  | (1.72E-5) | (2.84E-4) | (2.29E-4) | (3.45E-4) |
| Race and Ethnicity [Non-Hispanic White] |  |  |  |  |
| Non-Hispanic Black | -0.030*** | -0.039*** | -0.048*** | -0.081*** |
|  | (0.007) | (0.010) | (0.010) | (0.014) |
| Hispanics | 0.002 | -0.024** | -0.013 | -0.064*** |
|  | (0.005) | (0.010) | (0.010) | (0.015) |
| Non-Hispanic Asians and Others | -0.008 | 2.20E-4 | -0.011 | 0.020 |
|  | (0.008) | (0.013) | (0.016) | (0.022) |
| Married | 0.005 | 0.012 | -0.011 | 0.024* |
|  | (0.005) | (0.008) | (0.008) | (0.012) |
| Education [Less Than High School] |  |  |  |  |
| High School Graduate | -0.008 | -0.005 | -0.005 | 0.018 |
|  | (0.006) | (0.012) | (0.010) | (0.014) |
| Some College | 0.007 | 0.005 | 0.004 | 0.057*** |
|  | (0.006) | (0.012) | (0.010) | (0.015) |
| College Graduate and Above | 0.007 | 0.027** | 0.003 | 0.064*** |
|  | (0.007) | (0.012) | (0.011) | (0.016) |
| Worked in the Past 12 Months | 0.009* | 0.004 | 0.014* | -0.025** |
|  | (0.006) | (0.009) | (0.008) | (0.012) |
| Number of Adults in Family | -0.002 | 0.003 | 0.002 | -0.009 |
|  | (0.003) | (0.005) | (0.005) | (0.008) |
| Number of Kids in Family | 0.003* | -0.009*** | 0.007** | -0.012* |
|  | (0.002) | (0.003) | (0.003) | (0.006) |
| Covered by Any Health Insurance | -0.008 | 0.091*** | -0.015 | 0.159*** |
|  | (0.005) | (0.010) | (0.010) | (0.020) |
| Self-Reported Health Status [Excellent] |  |  |  |  |
| Very Good | 0.010** | 0.002 | 0.012 | 0.040*** |
|  | (0.005) | (0.008) | (0.011) | (0.015) |
| Good | 0.017*** | 0.011 | 0.027** | 0.055*** |
|  | (0.005) | (0.009) | (0.011) | (0.015) |
| Fair/Poor | 0.036*** | 0.028* | 0.059*** | 0.094*** |
|  | (0.008) | (0.015) | (0.012) | (0.016) |
|  |  |  |  |  |
| **Table S2: Marginal Effect of Factors Associated with Flu Vaccination and Infection (Cont'd)** | | | | |
|  |  |  |  |  |
| Variables | Low Priority Group | | High Priority Group | |
|  | Infection | Vaccination | Infection | Vaccination |
| BMI Group [Normal Weight] |  |  |  |  |
| Underweight | 0.029** | 0.003 | 0.002 | -0.023 |
|  | (0.012) | (0.027) | (0.025) | (0.036) |
| Overweight | 0.002 | 0.007 | 0.013 | 0.017 |
|  | (0.005) | (0.008) | (0.008) | (0.012) |
| Obese | 0.007 | 0.009 | 0.019** | 0.025* |
|  | (0.005) | (0.009) | (0.009) | (0.012) |
| Regular Place for Preventive Medical Care | -0.012** | 0.072*** | -0.018 | 0.158*** |
|  | (0.006) | (0.012) | (0.012) | (0.024) |
| Smoking Status [Current Smoker, Everyday] |  |  |  |  |
| Non-Smoker | -0.016*** | 0.047*** | -0.032*** | 0.069*** |
|  | (0.005) | (0.010) | (0.008) | (0.015) |
| Current Smoker, Sometimes | -0.009 | 0.032* | -0.022 | 0.065** |
|  | (0.009) | (0.018) | (0.017) | (0.029) |
| Drinking Status [Current Heavy Drinker] |  |  |  |  |
| Current Non-Drinker | 0.015 | 0.032** | -0.025* | 0.063** |
|  | (0.009) | (0.016) | (0.015) | (0.026) |
| Current Moderate Drinker | 0.011 | 0.019 | -0.027* | 0.088*** |
|  | (0.009) | (0.015) | (0.014) | (0.026) |
| Physical Exercise: >= 150 Min/Week | 0.003 | 0.017** | 0.010 | 0.008 |
|  | (0.004) | (0.007) | (0.008) | (0.011) |
| Strength Training: >= Once/Week | 0.004 | 0.020** | -0.003 | 0.064*** |
|  | (0.005) | (0.008) | (0.010) | (0.014) |
| Correlation: Pr(Infection) & Pr(Vaccination) | -0.067*** | | -0.361*** | |
| Rho (ρ) | -0.292 | | -0.741 | |
| Prob > chi2 | 0.000 | | 0.000 | |
| Observed Probability | 0.040 | 0.175 | 0.063 | 0.491 |
| Observations | 10,794 | 13,078 | 4,952 | 9,735 |
| Note: Average marginal effects are reported from Heckman probit selection model. The marginal effects of flu infection are conditional on being not vaccinated. The reported correlation is between predicted probability of flu infection conditional on being not vaccinated and predicted probability of flu vaccination. Rho (ρ) is the correlation between the error term of the selection equation and that of the outcome equation. Group in the brackets is the reference group. Significance: * p<0.10, ** p<0.05, *** p<0.01. | | | | |
|
|
|
|
|
